# Semiochemical applications for managing the grey field slug ( *Deroceras reticulatum* Müller), a major pest of arable crops

**DOI:** 10.1002/ps.70007

**Published:** 2025-06-23

**Authors:** Suleiman Mustapha, E Joel Loveridge, Tariq M Butt, Jozsef Vuts, Patricia A Ortega‐Ramos, Samantha M Cook

**Affiliations:** ^1^ Department of Biological Sciences Swansea University Swansea UK; ^2^ Protecting Crops and the Environment Rothamsted Research Harpenden UK; ^3^ Department of Chemistry Swansea University Swansea UK

**Keywords:** integrated pest management, mollusc, sustainable agriculture, repellents, molluscicides, push–pull

## Abstract

The grey field slug (*Deroceras reticulatum*) is a globally important gastropod pest of arable crops. The withdrawal of synthetic chemical molluscicides due to human health and environmental concerns has prompted research into other control methods. Particular attention has focussed on behaviour‐modifying chemicals (semiochemicals), most of which are derived from plants and other natural sources and include attractants, repellents and deterrents. This review provides a comprehensive overview of the use of semiochemicals in managing *D. reticulatum* and discusses their potential for use in integrated pest management (IPM) programmes. We reviewed trends in research publications on semiochemicals in relation to synthetic molluscicides and biological control methods. Besides the identification of promising plant‐based candidate semiochemicals, plants with attractant properties were identified for use as trap crops. These could be used with the main crop treated with repellents in ‘push–pull’ IPM programmes. Extracts from plants as well as predators and entomopathogenic fungi have shown promise against grey field slugs by inducing avoidance, antifeeding behaviour or even mortality, thereby reducing crop damage. Elucidation of the structure and mode of action of specific chemical compounds responsible for slug attraction or repulsion could lead to the development of new products for management of the grey field slug. © 2025 The Author(s). *Pest Management Science* published by John Wiley & Sons Ltd on behalf of Society of Chemical Industry.

## Graphical abstract

Trends in semiochemical research highlight promising attractants and repellents for ‘push–pull’ pest control strategies of the grey field slug. Extracts from plants, predators, entomopathogenic fungi, and essential oils demonstrate potential in modifying grey field slug behaviour, inducing avoidance, antifeeding responses, or mortality, offering sustainable alternatives to synthetic molluscicides.

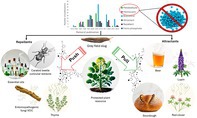

## 1 INTRODUCTION

Terrestrial molluscs (Pulmonata: Stylommatophora) are significant crop pests worldwide.^1^ The grey field slug, *Deroceras reticulatum* (Müller, 1774) (Agriolimacidae), is widespread globally, particularly across temperate regions.^2^, ^3^, ^4^, ^5^, ^6^, ^7^, ^8^, ^9^, ^10^ It is highly polyphagous and major agricultural crops such as wheat (*Triticum aestivum* L.), oilseed rape (*Brassica napus* L.),^9^, ^11^ soybean (*Glycine max* L.), alfalfa (*Medicago sativa* L.) and turnip (*Brassica rapa* subsp. *rapa*),^2^ are susceptible to *D. reticulatum* herbivory, especially during the establishment stages. *Deroceras reticulatum* can be challenging to manage considering the limited range of commercially available molluscicides.^12^ The prolonged use of molluscicides based on the active ingredients metaldehyde or methiocarb has resulted in significant environmental issues; in particular the pollution of surface water courses which is very difficult to mitigate.^9^, ^13^ This has led to legislative action for their withdrawal in the United Kingdom (UK).^9^, ^14^, ^15^, ^16^ Ferric phosphate is currently the only widely available conventional molluscicide approved in the UK, however its effectiveness is considered lower than that of metaldehyde, particularly for *D. reticulatum* when compared against other slug species.^17^ Achieving efficient levels of slug control therefore generally requires higher application rates, thereby increasing costs^17^ and the risk of resistance. The reliance on, and widespread use of products based on this active ingredient alone across large hectarages of a range of major agricultural crops in the UK is a growing concern. Available alternatives remain very limited,^12^ and inadequate slug control measures have been estimated to result in annual losses of over £100 million for farmers in the UK.^9^ This has prompted an urgent search for environmentally benign products and strategies.^9^, ^13^, ^18^

Attractive and repellent semiochemicals (naturally occurring behaviour‐ and development‐modifying compounds) that derive from plant or arthropod material and work at a distance,^19^, ^20^, ^21^ or deterrents/antifeedants and irritants that act upon contact,^18^ can be strategically used in push–pull integrated pest management (IPM) programmes to sustainably reduce pest damage.^20^ In push–pull systems, less attractive crop cultivars and/or repellent or deterrent semiochemicals are used to ‘push’ pests out of the cash crop while simultaneously ‘pulling’ them to attractive trap crops or crop areas supplemented with semiochemical attractants. Extracts and essential oils from legumes,^22^, ^23^ weeds,^11^, ^24^, ^25^, ^26^, ^27^, ^28^ ornamental plants and vegetables,^1^, ^5^, ^29^, ^30^, ^31^, ^32^ herbs^33^ and grassland plants^6^, ^34^ have been explored as potential sources of semiochemicals for *D. reticulatum* control.^35^ However, there has been insufficient follow‐up research to corroborate their effectiveness in field conditions, including species‐specificity.^36^ Furthermore, *D. reticulatum* has been shown to exhibit a heterogenous distribution across arable crop fields, with localised patches of high slug density that remain relatively stable throughout the growing season.^37^, ^38^ This spatial stability presents an opportunity for more targeted, low‐cost pest management approaches. The strategic application of semiochemicals could exploit these persistent distribution patterns, allowing for more efficient interventions.

This review synthesises existing knowledge on *D. reticulatum* olfaction and the use of semiochemicals for its management. It examines the advantages and limitations of experimental approaches, highlights gaps in previous research, and suggests future directions for developing more effective and sustainable slug control strategies.

### 1.1 Trends in research on control of *D. reticulatum*

**Figure 1:**
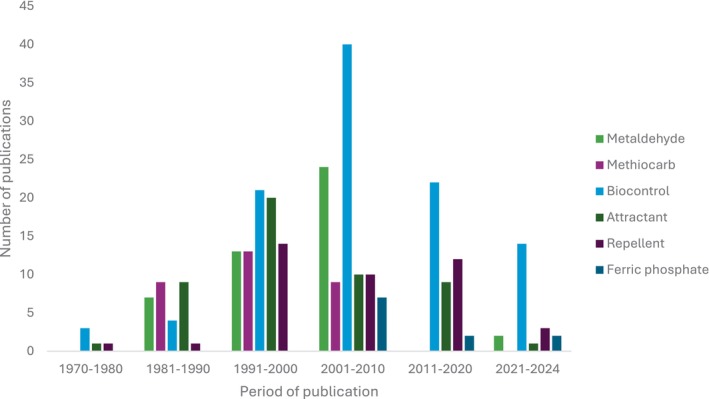
Trends in pest control studies on *Deroceras reticulatum*. Here, ‘repellent’ refers to studies that focused on antifeedants, repellents, essential oils and any compounds (apart from synthetic molluscicides) that act as molluscicides. ‘Attractants’ denote studies investigating plant‐based or ‐derived attractants and other products found to attract slugs (excluding synthetic molluscicide baits). ‘Biocontrol’ refers to studies examining the use of bio‐antagonistic agents, such as nematodes, carabid beetles, and pathogens.

In order to examine the trends in research and use of synthetic molluscicides, biocontrol, attractants and repellents, a systematic literature search was conducted using the Web of Science database. The following search word phrasing ‘*Deroceras reticulatum* and control’, ‘metaldehyde and *Deroceras reticulatum*’, ‘methiocarb and *Deroceras reticulatum*’, ‘iron phosphate and *Deroceras reticulatum*’, ‘ferric phosphate and *Deroceras reticulatum*’, ‘semiochemicals and *Deroceras reticulatum*’, ‘attractant and *Deroceras reticulatum*’, ‘deterrent/repellent and *Deroceras reticulatum*’, ‘grey garden slug’, and ‘grey field slugs’, yielded 2837 publications. From these, articles specifically addressing repellents, irritants, attractants, semiochemicals, botanicals and antifeedants were selected for detailed review and analysis. Out of the 2837 publications screened, only 239 studies were relevant to our analysis. A significant proportion of these publications focused on biological control of *D. reticulatum* (Fig. 1), indicating a trend towards intensifying research on biological control agents over synthetic molluscicides. This shift reflects efforts to promote sustainable pest management. Particularly interesting is the decline in studies on methiocarb and metaldehyde from 2011 to 2024, coinciding with the period of their withdrawal from use in the EU (2014) and the UK (2022), respectively, and the majority of the few studies on ferric phosphate emerging between 2021 and 2024. This limited study on ferric phosphate reveals a significant knowledge gap regarding its long‐term effectiveness, ecological impact and potential integration into sustainable pest management strategies, especially as pressures continue to restrict the use of synthetic alternatives. However, a research gap is also evident regarding the use of semiochemicals, i.e., repellents and attractants (Fig. 1), to control *D. reticulatum*. Relatively few studies have explored these potential tools, with the highest number of studies on both attractants and repellents recorded between 1991 and 2000, followed by a subsequent decline (Fig. 1). Further exploration in this area holds significant promise for developing innovative IPM tactics for *D. reticulatum* with lower environmental impact.

### 1.2 Life cycle of *D*. *reticulatum*

**Figure 2:**
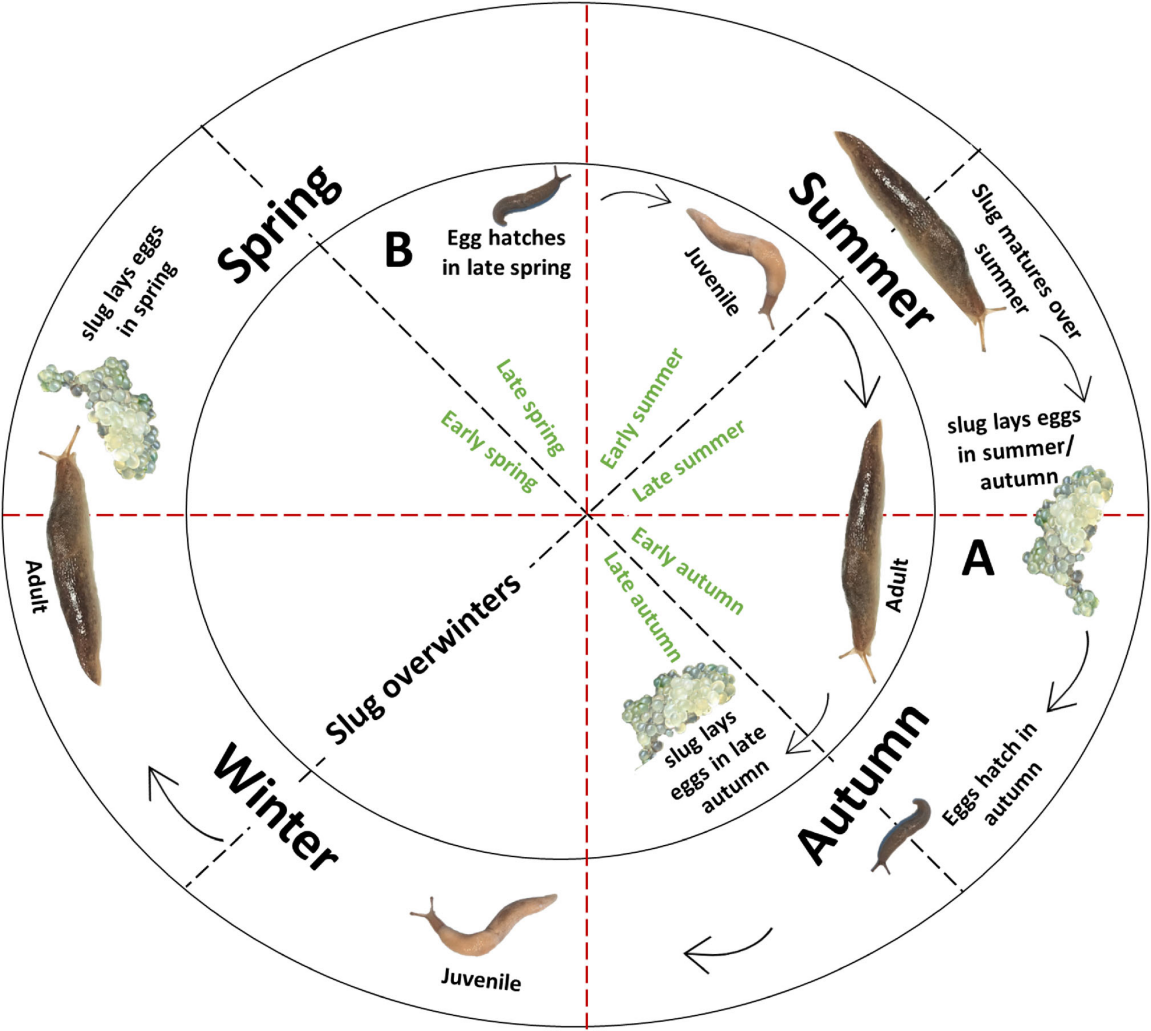
Illustrated life cycle of the grey field slug *Deroceras reticulatum*. Generation A and Generation B represent cohorts arising from autumn and spring egg hatchings, respectively, reflecting the species' overlapping life cycle.

*Deroceras reticulatum* are semelparous, typically univoltine species, producing one generation a year,^39^, ^40^ although bivoltine life cycles have also been observed under certain conditions.^41^ Their life cycle is affected by geographical, weather and climatic patterns, with extreme cold conditions slowing down their development.^39^, ^42^ Despite being a hermaphrodite species with the ability to self‐fertilise,^8^ they typically engage in copulation with other individuals for cross‐fertilisation.^8^, ^39^, ^43^ They can breed at any time of the year, producing approximately 200–500 small, round, translucent eggs which are usually laid in clusters^44^, ^45^, ^46^ (Fig. 2). Their life cycle from eggs to adults spans between 9 to 12 months.^39^, ^40^, ^47^ In temperate regions, *D. reticulatum* activity, unlike other slug species, typically peaks twice: in spring and again in autumn, driven by overlapping generations with usually a 9‐month interval. Eggs hatching in autumn result in slugs maturing over winter, which produce new eggs the following spring (Fig. 2A). Eggs hatching in the spring mature over summer, then lay eggs in late autumn (Fig 2B).^8^, ^39^ Adults can remain active during winter,^39^ but severe cold temperatures trigger the physiological state of ‘chill coma’ without being completely immobilised.^8^, ^48^

The life stages of *D. reticulatum* vary based on the season when the eggs hatch (Fig. 2), with two distinct types of development known as the slow and fast growth stages.^8^ During autumn and winter, eggs and juveniles develop slower than in warmer conditions. In cold conditions, eggs can take up to 5 months to develop, while the juveniles require up to 7 months or more to mature. However, in relatively warmer spring conditions, this might only take 2 to 3 months.^8^, ^39^.

## 2 SLUG OLFACTION

Semiochemicals play a significant role in aiding many terrestrial (pulmonated) gastropod species to locate and find food sources while foraging.^49^ Terrestrial gastropods possess one or two pairs of tentacles on their head, where both the eyes and olfactory epithelia are located.^49^ The tentacular structures play essential roles in olfaction.^50^, ^51^ Land slugs can learn and remember new information about scents, including those associated with negative olfactory stimuli.^52^ They can also differentiate novel olfactory cues from those they have encountered before and tend to show preference for certain scents.^49^, ^53^, ^54^, ^55^

The ability to detect and distinguish between odours is believed to be facilitated by a unique epithelial pad located at the ventral tip of the tentacle when outstretched.^51^ Olfactory detection in slugs is associated with the tips of two sets of tentacles, referred to as the inferior for the shorter ones (anterior) and superior tentacles for the longer ones (posterior), the latter also containing the eyes.^49^, ^52^ The primary olfactory organ of *D. reticulatum*, the superior tentacle, has two key nerves, the olfactory and the optic. They stem from the metacerebrum, a part of the cerebral ganglion, with the olfactory nerve ending in the digitate (tentacular) ganglion.^56^ Nerves in both sets of tentacles convey scent‐related information to the central nervous system, specifically to the procerebrum, for processing, memory formation and learning.^52^, ^57^ The procerebral lobe receives first‐stage input from olfactory receptor cells, as well as second‐stage input from the digitate ganglion.^53^, ^58^, ^59^

The highly developed olfactory system of slugs is very important for chemoreception involved in food finding, assessing the risk of predators and as a homing strategy to navigate back to specific locations.^60^, ^61^, ^62^, ^63^, ^64^, ^65^ The inferior and superior pairs of tentacles exhibit distinct functional roles in olfaction, with the superior tentacles more linked to orientation towards airborne volatile cues and the inferior tentacles for trail following or contact (gustatory) cues.^49^, ^51^, ^54^, ^56^ Furthermore, slugs use both pairs of tentacles to acquire olfactory cues, which are then recalled from their memories; this is observed even after surgical amputation on slugs previously conditioned with specific repellent odours.^50^, ^52^ The use of both pairs of tentacles to detect aversive odours might confer a survival benefit.^52^

## 3 METHODS TO EVALUATE SLUG BEHAVIOUR

A wide variety of methods have been used to study slug behaviour and orientation to chemical stimuli. Olfactory studies offer a means to assess the effectiveness of novel compounds in attracting slugs to bait in order to enhance their intake of molluscicides or to study their behaviour to repellents.^66^ However, it is important to validate laboratory assays with field trials to upscale results to realistic conditions.^67^ Here, we review some of the different techniques that have been used to study the olfactory behaviour of slugs.

### 3.1 Glass tube olfactometry assays

Commonly utilised to study the behavioural response of slugs to attractant or repellent odours are olfactory choice assays, where a sterile glass apparatus with at least two arms, typically T or Y‐shaped, is utilised, with the control and test samples placed at the ends of the different arms. A slug is then placed into the central stem and its movement observed, with a predetermined point in each arm indicating a choice.^10^, ^66^, ^68^ While most olfactometers are designed to deliver a constant flow of purified air to transport the test substances from the source point through the arms to reach the slug^69^ some slug species have been reported to be negatively anemotactic. They may not therefore necessarily move towards air flow, as they avoid windy interference.^68^, ^70^, ^71^, ^72^ In such cases still air olfactometer experiments may require larger sample sizes and extended time periods to detect a clear response.^68^ Olfactometer choice test experiments are relatively cheap and straightforward but slug behaviours regarding anemotaxis may lead to difficulty in drawing conclusions regarding the orientation preference of slugs towards chemical cues. Another design, technically more complicated but which overcomes these problems is a tentacular two glass chambered olfactometer, which delivers odours directly to the sensory tentacles, with independent adjustment capability over the levels of concentration to be administered on the right and left tentacle. Distinct head movements while the mollusc is moving, subject to latency and extent, suggest an orientation decision.^73^

### 3.2 Feeding bioassays

Feeding assays are very useful for providing insights into slug responses to close‐range stimuli such as stimulants and deterrents. They can be applied to both no‐choice and choice scenarios.^19^, ^72^, ^74^ In a choice assay, slugs are given an option between treated and untreated food samples, and their consumption is recorded at intervals to determine preference patterns. In a no‐choice assay, they are offered only the treated food sample and responses compared to a no‐choice standard treatment (control) to assess if the administered treatment changes their normal feeding behaviour.^19^, ^74^ Assays like this are often utilised to determine the effectiveness of different bait pellets in delivering toxins to slugs and could be extended to test the effect of different repellents and antifeedants *per se*. However in many studies, the plant materials used in feeding assays are usually presented as leaf discs, macerated or pelletised; processes that significantly alter the natural volatile profile of the plant.^75^ As a result, feeding bioassays may not accurately reflect real‐time interactions between slugs and living plants in the field.

### 3.3 Trail‐following bioassays

Trail‐following happens when one animal consistently traces the path of another, more often and for longer distances than would be expected by random chance.^76^ One method to understand trail‐following that has been proven effective to identify active volatile compounds attractive to *D. reticulatum* is the use of moistened filter paper with a drawn pencil line serving as trail reference, which is then placed in a black‐walled evaporating dish. The volatile test solutions are then applied along the pencil lines and the slugs introduced at the start of the line. After a specified period, the slugs are removed and their mucus trails visualised with the application of carbon powder. The mucus trail length is measured and compared to a control trail reference treated with deionised water.^68^ Other techniques have utilised transparent sheets (e.g. polythene substrate, acetate sheets, etc.) to study how molluscs track trails.^76^, ^77^, ^78^ These transparent materials are often dusted with a powder, for example charcoal,^78^ to make mucus trails visible, enabling researchers to observe and accurately quantify trail‐tracking behaviour.

The limitation of this technique is that the slug is made to have physical contact with the test compound and there is no analysis of their initial movement towards (or away from) the volatile source.^68^ Notwithstanding, understanding slug trails and movement patterns could improve their management.

### 3.4 Electrophysiological assays

In electrophysiological assays the main olfactory organ, the posterior tentacle, is dissected from an anesthetised slug and placed in a dish containing a specialised ringer solution. The tentacles are carefully dissected to expose both the sensory pad and olfactory nerve by removing the surrounding sheath tissue and muscles.^19^, ^56^ This setup exposes the nerve to the ringer solution while applying airborne volatile cues to the sensory pad. Electrical responses to the different volatile cues are recorded using a setup that involves suction electrodes, with an airstream to convey the test extract, and amplification equipment (for example DAM50 differential amplifier) to measure nerve activity. This technique has been successfully used to provide detailed examination of the response of the olfactory nerve of *D*. *reticulatum* to various chemical compounds and in the evaluation of olfactory functions in molluscs.^19^, ^56^, ^61^ However, the successful execution of electrophysiological recording requires highly specialised skills, which are often scarce.

### 3.5 Radio‐tracking bioassays

Recent studies have used radio‐tracking technology to monitor the movement patterns and behaviour of *D. reticulatum* in commercial crop fields, with individual slugs tagged using radio frequency identification (RFID) devices.^79^, ^80^ This method enables the unique identification and profiling of individual slugs, providing valuable insights into their spatial dynamics and activity.^79^, ^80^ To achieve this, slugs are anaesthetised using carbon dioxide (CO_2_) to induce full body extension. Next, RFID tags (a small glass capsule encasing both the chip and antenna coil), each bearing a unique identification code, are implanted beneath the body wall using an MK165 implanter.^79^, ^80^ The tagged slugs are released into the designated study field, where their movement patterns are monitored at predetermined intervals using a handheld portable reader. Each detected slug is identified by its unique RFID code, and its precise location and its activity (feeding, mating, etc.) is visually verified along with time stamp and spatial position.^80^

While most studies primarily rely on laboratory setups to test hypotheses, radio‐tracking offers a significant advantage by providing real‐time, field‐based insights into slug movement patterns, whether they are attracted to or repelled by sources of semiochemical cues. A potential drawback in the use of RFID tagging is that, although it has been shown not to affect feeding, locomotion or egg‐laying behaviour, improper insertion of the tag may alter their natural behaviour. This could lead to inaccurate observation and misinterpretation of movement patterns.

## 4 SLUG ATTRACTANTS

Slugs, due to their polyphagous nature,^2^, ^4^, ^6^, ^7^ are attracted to a range of plant metabolites.^68^, ^81^ Several attractants have been developed from plant extracts. These have been included in chemical lures, food bait, trap crops and in more traditional approaches like use of beer and fermented products as bait for slugs. Various attractants have been used successfully to entice different slug species (summarised in Table 1) however, this section will focus on *D. reticulatum*.

**TABLE 1:** Attractants for *Deroceras reticulatum* and other terrestrial mollusc species

Mollusc species	Chemical/product	Comment	References
*Deroceras reticulatum*, *Cornu aspersum*	Volatiles from chopped cucumber (*Cucumis sativus*).	Molluscs have inherent preference for cucumber.	66
*Deroceras reticulatum*, *Parmarion martensi, Ambigolimax valentianus*, *Cornu aspersum*, *Lissachatina fulica*, *Xerolenta obvia*	Fermenting bread dough	The fermentation process, especially with yeast, produces an aroma that attracts slugs, and the dough itself can be used as bait in traps or in attract‐and‐kill approach.	10
*Arion vulgaris,* *Deroceras reticulatum,* *Deroceras laeve*	Beer (fresh and stale) volatile fraction. Metaldehyde in bait with beer, Bay 37344 (4‐(methylthio)‐3,5‐xylyl methylcarbamate) with beer.	The volatile compounds, usually influenced by the type of yeast strain used to ferment beer, rather than the alcohol, are the main attractants.	82, 83, 84, 85, 86
*Deroceras reticulatum*	Malt beverage and malted grain fibre	Non‐alcoholic malt beverages and malted grain fibre as by‐product of brewery waste effectively attracted slugs, with increased attractiveness when supplemented with active yeasts and sucrose.	83
*Deroceras reticulatum*	(*Z*)‐3‐Hexen‐l‐ol	Vacuum distillation of lettuce leaves.	50
*Deroceras reticulatum*	Unfermented fresh grape juice, *Drosophila*‐fermented bait, bran bait containing 2% Bay 37344 (4‐(methylthio)‐3,5‐xylyl methylcarbamate) with beer, metaldehyde (4%) in corn cobs moistened with beer, metaldehyde 4% in corn cobs with water	Fresh unfermented grape juice moderately attracted slug. Commercial baits increased in effectiveness after being moistened with beer.	82
*Deroceras reticulatum*, *Arion lusitanicus*, *Arion rufus*	*Ocimum basilicum, Brassica napus* and *Coriandrum sativum*	Highly acceptable plant species to the molluscs.	87, 88
*Deroceras reticulatum*	Dandelion (*Taraxacum officinale*), carrot (*Daucus carota*) and lettuce (*Lactuca sativa*)	Volatile cues from plants attracted slugs.	68
*Deroceras reticulatum*, *Arion lusitanicus*, *Lehmannia nyctelia*	White clover (*Trifolium repens* and *Trifolium semipilosum*)	Very palatable to slugs. *Trifolium repens* is known to have high levels of hydrogen cyanide (HCN). Potentially could be a repellent but slugs are attracted to the forb species.	22, 34, 42, 88, 89
*Deroceras reticulatum*, *Arion lusitanicus*	*Capsella bursa‐pastoris*, *Stellaria media*, *Taraxacum officinale*, *Trifolium repens* and *Chenopodium album*	Common agricultural weed species palatable to slugs.	11, 24, 25, 26, 90
*Deroceras reticulatum*	Red clover (*Trifolium pratense*), lupin (*Luponus* spp.), alfalfa (*Medicago sativa*), white clover (*Trifolium repens*), vetch (*Vicia* spp.) and, birdsfoot trefoil (*Lotus corniculatus*).	When compared to winter wheat, with red clover being the most attractive.	22, 34, 91, 92, 93, 94, 95, 96, 97
*Deroceras reticulatum*, *Arion lusitanicus*	Cornflower (*Centaurea cyanus*)	Attractive to slug.	30
*Deroceras reticulatum*	Thin‐leaved rosette forest forb species *Aster cordifolius* and *Cryptotaenia canadensis*.	Palatable to slug.	33
*Deroceras reticulatum*, *Arion rufus*, *Arion vulgaris*	The sweet lupin cultivar regent and bojar	Lupins with low alkaloid contents are highly attractive to slugs.	98
*Deroceras reticulatum*, *Arion lusitanicus* and, *Deroceras agreste*	*Arnica montana*	All three species of slug preferred undamaged *A. montana* leaves when compared to 20 other meadow plant species.	99
*Deroceras reticulatum*	*Sonchus arvensis* (perennial sow thistle), *Cichorium intybus*, *Leontodon hispidus*	Palatable to slug.	100
*Deroceras reticulatum*, *Arion hortensis*, *Arion distinctus*	Brussel sprouts and lettuce	Slugs are attracted to Brussel sprouts.	5

### 4.1 Plant attractants

The use of attractive ‘trap’ plants as an alternative food source may effectively divert slugs away from the primary crop.^11^ In fact, providing a diversity of plants (e.g., wildflowers) alongside crop fields could offer protection of the main crop by reducing the pressure of mollusc herbivores.^101^

Several weed species commonly found within agricultural fields have been identified to have potential to serve as readily‐available alternative food source for slugs.^11^, ^24^, ^25^, ^68^, ^102^ Slugs are attracted by the volatiles released by these plants,^49^, ^68^, ^72^ which can be leveraged as a ‘pull’ strategy within a push–pull IPM approach by luring slugs away from the main crop during periods of establishment. Growing weed species like shepherd's purse (*Capsella bursa‐pastoris*) and chickweed (*Stellaria media*) alongside oilseed rape has shown promise in reducing slug feeding on the crop, with effectiveness comparable to molluscicide pellet bait.^11^, ^26^ However, this effect has only been observed under low slug population density.^11^ A hierarchy of preference of *D. reticulatum* to dandelion (*Taraxacum officinale*), shepherd's purse, white clover (*Trifolium repens*) and fat hen (*Chenopodium album*) as most preferred when compared alongside 12 winter wheat cultivars was observed.^24^ Nevertheless, growing weeds alongside crops, while potentially favourable for slug management, has the obvious disadvantage of more competitive weed populations.^12^ Therefore, sustainable weed management practices are crucial to balance the benefits of using weeds as slug attractants while preventing the consequence of exacerbating weed problems in agricultural fields. However, the specific attractive properties of these weed species have not been fully identified. A potential strategy for effectively utilising weed species is to isolate and deploy the slug attractants found in these without introducing or promoting additional weed development.^103^

Legumes could be utilised as trap crops to mitigate slug infestations during crop establishment, with red clover (*Trifolium pratense*), lupin (*Lupinus perennis*), lucerne (*Medicago sativa*) and white clover demonstrating high attractiveness to *D. reticulatum*, compared to winter wheat.^22^, ^34^, ^97^, ^98^ Notably among these legumes, red clover returned the greatest reduction in feeding damage (50–78% decrease in the mean amount of wheat consumed), indicating its strong potential to be used as a trap crop to divert *D. reticulatum* away from the main crop.^22^, ^94^, ^96^, ^97^ However, the drawback of growing red clover in the same plot as winter wheat is that it can negatively impact wheat yield (by up to 43%, compared to wheat plots without red clover).^97^ This suggests that while red clover may be an important trap crop to protect wheat during its vulnerable seedling stage, it must be removed from the field once the wheat has fully established to avoid yield loss.^97^ Best practice could be to grow red clover in designated strips of land within or adjacent to the main crop to serve as a pull to slugs. Similarly, the sweet lupin (*Lupinus angustifolius*) cultivars Regent and Bojar are highly attractive to *D. reticulatum*, while cultivars such as Mirela, Oskar and Karo containing high levels of lupanine alkaloids are less attractive. However, slugs may still consume these plants in the absence of alternatives, because they can detoxify secondary metabolites when present in non‐lethal concentrations, most probably via the cytochrome P450 enzyme, with younger slugs showing higher tolerance compared to older individuals.^98^, ^104^ *Arion vulgaris* slugs injected with non‐lethal doses of harmaline, sparteine, cytisine, lupanine, senecionine, quinidine and eserine could detoxify the alkaloids within 72 h, but higher concentrations killed them.^98^, ^104^

A study examining feeding damage by *D. reticulatum* on seedling monocultures of 23 meadow species and oilseed rape as reference identified a hierarchy of acceptability. Red fescue (*Festuca rubra*), Yarrow (*Achillea millefolium*), rough bluegrass (*Poa trivialis*) and Yorkshire fog (*Holcus lanatus*) were found to be more attractive to slugs than oilseed rape.^6^ This suggests that creating meadow buffer zones could be a viable strategy for crop protection against slugs, especially for oilseed rape as major crop. *Deroceras reticulatum* was found to be highly attracted to blue wood aster (*Aster cordifolius*) and Canadian honewort (*Crypotaenia canadensis*) forest herbs, consuming more of the plant leaves compared to others.^33^ Similarly, the perennial herb wolf's bane (*Arnica montana*) was highly attractive and palatable to three different slug species (*Deroceras agreste*, *Arion lusitanicus* and *D. reticulatum*).^99^

Many of the studies investigating slug attractants have, however been conducted under controlled microcosm conditions, which may not fully capture the complexities and variability of real‐world field environments. Factors such as weather fluctuations, soil heterogeneity, plant developmental stages and the spatial and temporal dynamics of slug density and distribution in the fields can significantly influence the attraction responses and long‐term effects of slug herbivory on plant populations. Furthermore, while various plant species have been identified to attract slugs, unfortunately most studies have not isolated or identified the specific chemical compounds responsible for the attraction, leading to a great dearth of information in this area. Future research should encompass a more detailed analysis using gas chromatography coupled to mass spectrometry alongside olfactory bioassays and electrophysiology to identify attractants.

### 4.2 Beer and other fermented products as attractants

Beer is an alcoholic drink produced by the brewing and fermentation of starches from cereal grains; the yeast converts sugar into alcohol or organic acid.^105^ Beer, poured into traps on the ground has long been used as a traditional method of slug control in domestic situations; slugs are lured and killed in the traps by drowning. Beer has proven to be a consistently effective attractant for slugs in both laboratory and field experiments. Various volatile compounds associated with beer have been identified as being attractive to slugs, including dihydroxyacetone, acetoin and diacetyl.^83^, ^86^, ^106^ However, the degree of attraction may differ depending on the brand and its chemical composition as well as species.^83^, ^84^, ^85^ Beer (both fresh and stale) was shown to be a potent attractant for *D. reticulatum* (Table 1) when compared to ethyl alcohol, methyl alcohol, 10% vinegar in water, 5% dimalt in water, dry grape wine, blackberry wine, and water control.^82^ Slugs apparently did not intentionally move into the liquid; instead, they slipped or slid in from the edges of the containers. Expanding on their work, Smith & Boswell dipped common commercial metaldehyde pellet baits in beer, which increased the effectiveness of the bait. However, the baits with beer did not outperform beer alone as attractant. Additionally, mixing beer with bran bait containing 2% of Bay 37344 (4‐(methylthio)‐3,5‐xylyl methylcarbamate) greatly improved its effectiveness in controlling slugs.^82^ It is worth noting that the attraction of slugs to beer is not due to its alcoholic content but rather the volatile compounds it contains.^82^, ^83^, ^86^, ^107^ In fact, non‐alcoholic beverages, such as malt drink, elicited a stronger attraction response of *D. reticulatum* when compared to a particular brand of beer.^83^ However, the mechanism driving slug attraction to beer is unclear. It is not yet fully understood whether attraction is primarily mediated by volatiles associated with the fermentation processes, sugar content, or the specific yeast strains used; different yeast strains significantly influence *D. reticulatum* attractiveness to beer likely due to the variations in volatile compounds they produce.^83^

While the use of liquid beer bait has considerable potential for managing *D. reticulatum*, its practical implementation may lead to increased crop protection costs, particularly in large‐scale arable systems. In addition, beer bait could attract non‐target organisms, including beneficials such as wasps that also have an ecological role in pest management.^108^ Therefore, further research is recommended to identify the specific chemical compounds responsible for eliciting slug responses to beer, a knowledge gap that clearly remains unaddressed to date. This could inform the development of species‐specific synthetic baits for use in IPM strategies while minimising unintended effects on non‐target organisms.

Other products of fermentation, like the use of fermenting bread dough formulations composed of water, sugar, flour and yeast, have been demonstrated to effectively attract different mollusc species with a maintained efficiency for at least 8 days, significantly outperforming common metaldehyde‐based baits.^10^ Additionally, fermenting sugar yeast (*Drosophila* bait) and fresh unfermented grape juice also act as moderate attractants for *D. reticulatum*.^82^ Fermentation products could therefore be used as part of attract‐and‐kill strategies or in baited traps to control slug populations, as they are usually non‐toxic, widely accessible and cost‐effective.^10^ Studies have shown that odours produced during fermentation, when sugar water is combined with yeast, can significantly attract slugs. Additionally, brewery by‐products like malted grain fibre exhibited considerable attractiveness to slugs, with the attraction being notably enhanced when supplemented with sucrose and active yeast.^10^, ^83^ With its simple composition and ease in acquiring the ingredients, the use of fermented products holds considerable potential for broader adoption in pest management practices, although they are mostly suitable for small‐scale applications. Nonetheless, further research is needed to identify the most consistent and stable chemical compounds emitted from fermented products that are highly attractive to slugs.

## 5 SLUG REPELLENTS, ANTIFEEDANTS AND MOLLUSCICIDES

Plants produce chemical compounds which act in defence against pests.^109^ For example, brassicas produce glucosinolates that can reduce generalist herbivore damage.^110^ Glucosinolates tend to occur in lower concentrations in agricultural brassica crops compared to wild relatives because they have been selectively bred for human consumption.^111^ Similar to other invertebrate herbivores, *D. reticulatum* feeding triggers anti‐herbivore defences in plants by activating specific defence signalling pathways (e.g., salicylic acid, jasmonic acid and abscisic acid). This activation enhances the plant's resistance to subsequent herbivory.^109^ In *Solanum dulcamara*, these defences involve the production of various secondary plant metabolites and volatile organic compounds (VOCs). The key compounds identified after *D. reticulatum* herbivory on solanum include glycoalkaloids, anthocyanins, phenolamides (*N*‐caffeoylputrescine), as well as polyphenol oxidase and trypsin protease inhibitors.^109^ These compounds collectively contribute to anti‐herbivore defence. In this section, we review known repellents that direct slug movement away from their source, and antifeedants, which reduce or prevent slug feeding damage upon contact. These compounds have been studied for their effects against *D. reticulatum* and are listed in Table 2. However, due to the heterogenous distribution of *D. reticulatum* within fields,^37^, ^38^ deploying repellents/deterrents in a push–pull context may only provide effective protection within actual slug‐infested patches. Therefore, research which enables targeting areas with high slug densities would enable spatially efficient placement of treatments thereby minimising active ingredient use and costs to effectively suppress *D. reticulatum* populations.

**TABLE 2:** Repellents, antifeedants and molluscicides identified from research that show potential for control of *Deroceras reticulatum* and other terrestrial mollusc species

Mollusc species	Chemical/product	Comment	References
*Deroceras reticulatum*	Cuticular extracts derived from the insects: *Carabus coriaceus*, *Musca domestica*, *Carabus auratus*, *Carabus nemoralis*, *Carabus hispanus* and chemical cues from *Pterostichus melanarius*	Chemical cues from carabid beetles impede slug foraging and force avoidance behaviour	61, 112, 113, 114, 115
*Deroceras reticulatum*	(+)‐Fenchone	It reduced feeding. Fennel (*Feoniculum vulgare* (Apiaceae)) contains significant levels of (+)‐fenchone	116, 117, 118
*Deroceras reticulatum*	γ‐Coniceine	Hemlock (*Conium maculatum*) contains large amounts of the alkaloid coniceine, which has antifeedant properties	19, 118
*Deroceras reticulatum*	Cinnamamide	Wheat seed dressing, acting as a repellent	119
*Deroceras reticulatum*	Vulpinic acid	Antifeedant metabolites extracted from the lichen *Letharia vulpine*.	120
*Deroceras panormitanum*, *Oxyloma pfeifferi, Deroceras reticulatum*	Cinnamamide, copper ammonium carbonate, urea formaldehyde, copper impregnated mattings, garlic concentrates, copper foil	Cinnamamide seed coating on winter wheat protected seeds from slugs. Did not affect viability of seeds. Garlic, urea formaldehyde and cinnamamide solution repelled slugs and caused over 95% mortality and protected crop from damage.	31, 32, 119, 121, 122, 123
*Deroceras reticulatum*	Extracts from the plants *Crithmum maritimum, Conium maculatum* (alkaloid coniceine), *Coriandrum sativum*, *Petroselinum crispum* and *Anthriscus cerefolium*	Significantly reduced *D. reticulatum* feeding	56
*Deroceras reticulatum*	Thyme, spearmint, and pine	The essential oil emulsions sprayed on plants with slugs in them caused significant level of mortality comparable to commercial molluscicides.	7
*Deroceras reticulatum*	Methanol extract from tarragon (*Artemisia dracunculus*), peppermint, rosemary Mexican tea plant and anise	Tarragon gave the highest antifeedant effect	118
*Deroceras reticulatum*	Geraniol	A potent feeding deterrent, pellets containing geraniol elicited a strongly negative feeding response.	116
*Deroceras reticulatum*	Glucosinolates	Oilseed rape variety with high levels of glucosinolates deterred slugs from feeding	110, 124
*Deroceras reticulatum*	Lolitrem B	Indole diterpenoids extracted from the endophytic Clavicipitaceous fungi genus *Neotyphodium* reduced slug feeding	125
*Deroceras reticulatum*, *Arion distinctus*, *Arion vulgaris*	Digested organic matter	Organic material that has undergone anaerobic digestion as part of biogas production process is strong repellent and molluscicidal to slugs	126
*Deroceras reticulatum*	Garlic mustard (*Alliaria petiolata*)	Slug avoided consuming this species in laboratory assays. *Alliaria petiolata* is known to contain cyanide as part of its chemical defence.	33
*Deroceras reticulatum*, *Arion rufus* and, *Arion vulgaris*	Alkaloids: lupanine, saparteine, atropine, quinidine, cytisine, senecionine, harmaline, eserine.	Slugs exhibited reduced feeding preference for the *Lupinus angustifolius* cultivars Karo, Oskar and Mirela with high alkaloid content and also avoided the feeds containing the alkaloids.	98, 104
*Deroceras reticulatum*	Saponin	Saponin extract from *Quillaja saponaria*, *Camellia oleifera* and *Gleditsia amorphoides* gave antifeedant and molluscicidal effects against slugs.	127
*Deroceras reticulatum*	4‐Pentenyl isothiocyanate	Elicited a repellent response at 0.1 and 1.0 μg/μL.	128
*Deroceras reticulatum*	Cedarwood oil and cedarwood in combination with *Phasmarhabditis hermaphrodita* a parasitic nematode.	Cedarwood oil caused mortality of the slug and was highly potent with the combination of parasitic nematodes.	36
*Deroceras reticulatum*, *Deroceras laeve*, *Arion subfuscus*	Phenolic glycoside: 6‐hydroxy‐l,2,3,4‐tetrahydro‐*β*‐carboline‐3‐carboxylic acid (6‐HTβC‐3‐COOH)	Quackgrass (*Agropyron* repens) extract fraction containing phenolic glycosides indicated both gastrointestinal and dermal toxicity	129
*Deroceras reticulatum*	Salosonine glycoalkaloid, anthocyanins, phenolamides (e.g., *N*‐caffeoylputrescine), polyphenol oxidase, trypsin protease inhibitors	Metabolites emitted from *Solanum dulcamara* after slug feeding increased plant resistance against further slug herbivory.	109, 130
*Deroceras reticulatum*, *Helix apersa*, *Arion hortensis*	Extracts from the myrrh plants opoponax (*Commiphora guidotti*) and (*Commiphora molmol*). The former containing both monoterpenes (*trans*‐*β*‐ocimene) and sesquiterpenes and the latter sesquiterpenes and furano‐sesquiterpenes.	The myrrh extract induced strong antifeeding behaviour at 0.5 and 1% to slugs but higher for snail (3–5%), also caused both repellence and mortality and performed better as natural physical barrier when compared to a top commercial product. Lettuce leaves treated with *trans*‐*β*‐ocimene caused 100% mortality.	131, 132
*Deroceras reticulatum*, *Arion fasciatus*, *Bradybaena fruticum, Arianta arbustorum*.	Aqueous suspension of crushed conspecifics	Deterred slug feeding and induced avoidance where the suspension was sprayed on crop	133
*Deroceras reticulatum*	Silicon (Si)	Increasing the foliar Si concentration in wheat seedling reduced grazing by slug.	134
*Deroceras reticulatum*	*Ximenia americana* (leaves and bark), *Detarium microcarpum* (bark) and, *Polygonum limbatum* (shoot).	The plants acted as effective repellents to slugs when used as barriers. Additionally, both alcoholic and aqueous extracts of the plants exhibited molluscicidal effects on slugs upon direct contact.	135
*Deroceras reticulatum*, *Arion ater*, *Deroceras laeve*, *Veronicella cubensis*, *Zonitoides arboreus*	Caffeine from spent coffee ground.	Spent coffee ground applied as top dressing on tomato and radish crop promoted plant growth and reduced slug herbivory by repelling them. Caffeine also acted as molluscicide.	136, 137
*Deroceras reticulatum*, *Cornu aspersum*	Volatile organic compounds (VOCs) from the fungus *Metarhizium brunneum* 1‐octen‐3‐ol, 3‐octanone and, 1‐octene.	The VOCs were repellent to both slug and snail at low dosage (1–5 μL) and caused death upon contact or as fumigant at higher dosage (10 μL).	74
*Deroceras reticulatum*	Neem and olive oil	Had ovicidal effects on slug eggs.	138, 139

### 5.1 Scents and cuticular extracts from predators as repellents

Avoidance of certain plants and animals suggest that the semiochemicals from them function as slug deterrents, antifeedants or as toxins when ingested.^56^ While most repellents are derived from plants, cues from predators have also been indicated to initiate avoidance. For instance, the scent of predatory carabid beetles has been demonstrated to modify the behaviour of slugs causing them to cease or reduce feeding^113^, ^114^, ^115^ (Table 2). Furthermore, adult carabids produce a distinctive odour attributed to the secretions from their defensive pygidial glands,^61^ which triggered notable olfactory responses in *D. reticulatum* ^61^ and caused it to flee upon detecting these cues.^113^ The compounds responsible for eliciting evasive response in slugs could be identified and used in crop protection but there is a lack of sufficient follow‐up studies to evaluate feasibility in field settings. Furthermore, the long‐term effectiveness of their use in the absence of predators is doubtful, as slugs might eventually become habituated to the cues over time.

### 5.2 Volatile organic compounds from entomopathogenic fungi as repellents

VOCs isolated from the conidia of the entomopathogenic fungus *Metarhizium brunneum*, specifically 1‐octen‐3‐ol, 3‐octanone and 1‐octene, repel the molluscs *Cornu aspersum* and *D. reticulatum* (Table 2). These compounds induce repellent behaviour at lower concentrations and act as fumigants or contact molluscicides at higher concentrations, ultimately killing the slugs.^74^ They might therefore hold promise for the development of new sprayable formulations to protect plants from mollusc attacks. However, these studies were conducted in controlled environments and results may differ from those in real‐world scenarios, particularly when evaluating long‐term effects.

### 5.3 Plant extracts as antifeedants

Certain plants or their extracts have the potential to function as antifeedants against *D. reticulatum*.^19^ Some compounds with antifeedant properties, for example (+)‐limonene, do not repel, demonstrating the need to distinguish between antifeedant and repellent modes of action.^19^ Antifeedant effects of certain compounds are primarily caused by their secondary metabolite content^120^ (refer to Table 2 for a full list of compounds). For instance, extracts derived from the lichen *Letharia vulpine* containing vulpinic acid as a main chemical component successfully deterred *D. reticulatum* from feeding when used as seed dressing on wheat or foliar spray on turnip plants.^120^

The monoterpenoid ketone (+)‐fenchone, extracted from fennel (*Feoniculum vulgare*), triggered a strong, enantiomer‐specific antifeedant behaviour from *D. reticulatum*.^116^, ^117^, ^118^ Geraniol has also been described as an effective antifeedant to slugs, causing up to 83% reduction in feeding.^116^, ^118^ However, its poor persistence and high volatility significantly hinder its practical application in field settings.^116^

Tarragon (*Artemisia dracunculus*) extract reduced feeding by 82% by *D. reticulatum*, followed by peppermint (*Mentha piperata*) (68%) and rosemary (*Rosemarinus officinalis*) extract (60%), whereas Mexican tea plant (*Chenopodium ambrosioides*) and anise (*Pimpinella anisum*) had lower antifeedant activity with 42% and 40% reduction, respectively.^118^

Of the 33 Apiaceae species screened for their antifeedant effect on *D. reticulatum*, extracts from rock samphire (*Crithmum maritimum*) and hemlock (*Conium maculatum*) reduced *D. reticulatum* feeding by 60–80%, while coriander (*Coriandrum sativum*), parsley (*Petroselinum crispum*) and chevril *Anthriscus cerefolium* caused a decrease in feeding behaviour by over 40%.^56^ Interestingly, exposing the tentacular nerve preparation of the slug to extracts elicited intense electrical activity, suggesting that *D. reticulatum* possess the ability to detect and discriminate these extracts.^56^ Furthermore, the alkaloid coniceine, derived from the hemlock plant, reduced *D. reticulatum* feeding by approximately 72%, suggesting it to be a potent antifeedant.^19^, ^118^

Although extracts from edible plants or from those widely used in therapeutics, nutraceuticals, cosmetics, and so forth are considered safe for use in crop protection programmes,^129^ it is important to recognise that some plant extracts can be toxic or cause adverse effects in humans and other non‐target organisms, depending on doses and species.^140^, ^141^, ^142^ In addition, certain extracts may be phytotoxic if applied above a specific concentration and may affect certain crop species or cultivars differently.^143^ Therefore, caution is necessary when testing these extracts for use in crop protection.

### 5.4 Plant defence toxins as antifeedants and molluscicides

*Deroceras reticulatum* tends to prefer brassicas with lower levels of glucosinolates over those with higher concentrations.^124^ However, it is important to note that higher concentrations of glucosinolates do not completely deter the pest from feeding on treated plants in the absence of alternative food sources. Saponin extracted from *Quillaja saponaria* (Soap‐Bark Tree), *Camellia oleifera* (tea‐oil camellia) and *Gleditsia amorphoides* were antifeedant and molluscicidal against *D. reticulatum* when administered orally within a concentration range of 1–4% *w/w*.^127^ The extracts exhibited potent membranolytic properties, leading to severe damage to the gastric epithelium of the crop region and as such could serve as a potential molluscicide. Although administration of the extracts was orally forced in the study, the use of pellet baits with phagostimulants could offer a practical means to entice slugs to consume the extract in field settings.

### 5.5 Multifunctional effects of certain chemicals and plants

Garlic, urea formaldehyde and cinnamamide have all been reported to exert multiple effects against snails and slugs, including acting as irritants, antifeedants, molluscicides and repellents.^31^, ^123^ In laboratory bioassays, garlic and cinnamamide demonstrated high repellence and induced mortality rates of up to 95% in *D. panormitanum*, a species closely related to *D. reticulatum*, and in the snail *Oxyloma pfeifferii*. This led to a significant reduction in crop plant damage ranging between 41% to 100%.^31^ However, further research to validate these findings in field situations and to understand the impact when applied on different crops is needed.

One significant challenge with utilising some of these compounds is their potentially high production costs, which can make them unsuitable for practical application, especially in commercial farms. Additionally, these products may not always meet customer needs or gain acceptance, posing further obstacles to their widespread adoption. Consequently, their application might be limited to very small‐scale horticulture.^31^

### 5.6 Molluscicidal potential of plant essential oils

Essential oils and their constituents have emerged as promising alternatives to synthetic molluscicides for the control of slugs,^7^, ^127^ the fumigant and contact toxicity of the oils offer versatile approaches for managing slugs. Although a comprehensive review of essential oils and their potential as molluscicides for gastropods is available,^35^ limited attention is directed towards compounds specifically targeting *D. reticulatum*. Potent molluscicidal effects of thyme (*Thymus vulgaris*), spearmint (*Mentha spicata*) and white pine (*Pinus strobus*) essential oils (Table 2) were shown when compared to cinnamon cassia, lemongrass, rosemary, garlic and peppermint.^7^ Their efficacy was comparable to chemical molluscicides, but crucially, they did not have any phytotoxic effects on the target crop at either seedling or matured plant stage. Additionally, there were no observed changes in plant biomass or chlorophyl content. Therefore, under persistent mollusc pressures in the field, these oils could be effectively delivered on to seedlings in spray formulations.

Essential oils show promising potential as ovicides for targeting the eggs of terrestrial molluscs. Of the 12 different essential oils/components (rosemary, white pine, d‐limonene, peppermint, spearmint, garlic, lemongrass, cedarwood, cinnamon, bitter orange, eucalyptus, and clove bud) screened, all but bitter orange, eucalyptus and d‐limonene caused mortality of the mollusc *Cornu aspersum* eggs and emerging juveniles at 1% concentration.^144^ Furthermore, eggs within infested media did not hatch as a result of treatment with clove bud oil, compared to control pots that had 100% emergence. Similarly, application of neem and olive oil caused mortality of *D. reticulatum* eggs, acting as an ovicide.^138^, ^139^ Therefore, oils could be used as a drench to target mollusc eggs; however, this approach is likely more viable for small‐scale horticulture due to the high cost of oils.^144^

The effectiveness of essential oils as repellents or molluscicides may vary depending on the species involved. Azadirachtin extracted from neem seeds (*Azadirachta indica*) negatively affects the probing and feeding behaviour of the cereal aphids *Sitobion avenae* (F.) and *Rhopalosiphum padion* (L.) on winter barley seedlings.^145^ However, the same extract had no effects on the slug species *D. reticulatum*, *Deroceras invadens* and *Arion distinctus*, indicating the species‐specific activity of azadirachtin. Also, neem oil is less effective against *D. reticulatum* compared to metaldehyde molluscicides.^146^ Cedarwood oil alone or in combination with the parasitic nematode *Phasmarhabditis hermaphrodita* exhibited significant repellent and molluscicidal effects against *D. reticulatum* and prevented crop damage, but it had no impact on *Arion vulgaris*.^36^ Another benefit of using cedarwood oil lies in its compatibility with biological control agents such as parasitic nematodes, unlike some other essential oils which can negatively impact nematode survival.^36^, ^147^ Essential oil extract from the oleoresin of the myrrh plant (*Commiphora guidotii*) opoponax and the active ingredient *trans‐β*‐ocimene caused significant repellence to *D. reticulatum* for up to 14 days after application, negatively affected their feeding behaviour and caused mortality without affecting non‐target organisms such as earthworms.^132^

One notable advantage of using essential oils is that they are usually exempted from legislative limits and pesticide residue requirements when compared to the use of synthetic pesticides. This exemption can significantly accelerate the regulatory approval process for essential oil‐based molluscicides, paving the way for quicker introduction into the pesticide market.^7^, ^18^, ^144^ Nevertheless, essential oils may negatively affect non‐target organisms if their concentration exceeds a certain tolerance limit.^142^ For instance, essential oils derived from cinnamon, pine, clove bud, peppermint, garlic, eucalyptus and lemongrass have been shown to exert moderate to high levels of toxicity against *Phasmarhabditis hermaphrodita*, a slug parasitic nematode vital for their biological control, as well as against *Steinernema feltiae*, an entomopathogenic nematode important for the control of pest insects.^35^, ^147^ Additionally, the use of essential oils may adversely affect generalist insect predators that contribute to natural pest suppression in agroecosystems.^148^ Therefore, further research is necessary to elucidate the broader ecological impacts of essential oils, and their use should be approached with caution to minimise unintended harm to beneficial organisms.

## 6 CONCLUSION

The polyphagous terrestrial mollusc *D. reticulatum* presents significant challenges in agriculture by causing substantial damage to economic crops, especially during plant establishment. Understanding and leveraging their attraction to and/or avoidance of specific chemical cues could lead to sustainable management alternatives for synthetic molluscicides. Attractive plants, such as red clover, have a strong potential to serve as trap crops by planting them in strips within fields or on edges to act as a pull factor, pulling slugs away from the main crops. Fermentation products such as beer, brewery by‐products, bread dough, and so forth have shown effectiveness as strong slug attractants. Elucidating the specific attractive volatiles could pave the way for the development of novel synthetic semiochemical lures for use in lure‐and‐kill strategies or as toxic baits. Repellents derived from natural sources offer a complementary technique for slug management especially as a push strategy to deter infestation of newly‐sown crops. Volatile compounds from slug predators, entomopathogenic fungi, and essential oils have potential for development as antifeedants and molluscicides, thereby directly reducing crop damage.

This review has highlighted that there is still a significant knowledge gap especially in the identification of species‐specific compounds; future efforts should therefore focus on this. Additionally, translating promising laboratory findings to the field scale is essential to validate their effectiveness in commercially‐relevant scenarios, as is testing their dose‐dependent impact on non‐target organisms. The integration of diverse semiochemical‐based approaches in push–pull or lure‐and‐kill strategies holds great potential for environmentally benign management of gastropod pests.

## FUNDING INFORMATION

This work was supported by the Biotechnology and Biological Sciences Research Council (BBSRC) – funded South West Biosciences Doctoral Training Partnership (SWBio DTP) [BB/T008741/1] (SM). SMC, JV and PAO‐R acknowledge support from the Growing Health Institute Strategic Programme [BB/X010953/1; BBS/E/RH/230003A].

## CONFLICT OF INTEREST

The authors declare no conflict of interest exists in the publication of this work.

## Data Availability

Data sharing is not applicable to this article as no new data were created or analyzed in this study.
